# High motor variability in DYT1 dystonia is associated with impaired visuomotor adaptation

**DOI:** 10.1038/s41598-018-21545-0

**Published:** 2018-02-26

**Authors:** Anna Sadnicka, Anna Stevenson, Kailash P. Bhatia, John C. Rothwell, Mark J. Edwards, Joseph M. Galea

**Affiliations:** 1 Sobell Department for Motor Neuroscience and Movement Disorders, Institute of Neurology, University College London, 33 Queen Square, London, WC1N 3BG UK; 2 Motor Control and Movement Disorder Group, Institute of Molecular and Clinical Sciences, St George’s University of London, Cranmer Terrace, Tooting, London, SW17 0RE UK; 3 Galea Lab, School of Psychology, University of Birmingham, Birmingham, B15 2TT UK

## Abstract

For the healthy motor control system, an essential regulatory role is maintaining the equilibrium between keeping unwanted motor variability in check whilst allowing informative elements of motor variability. Kinematic studies in children with generalised dystonia (due to mixed aetiologies) show that movements are characterised by increased motor variability. In this study, the mechanisms by which high motor variability may influence movement generation in dystonia were investigated. Reaching movements in the symptomatic arm of 10 patients with DYT1 dystonia and 12 age-matched controls were captured using a robotic manipulandum and features of motor variability were extracted. Given that task-relevant variability and sensorimotor adaptation are related in health, markers of variability were then examined for any co-variance with performance indicators during an error-based learning visuomotor adaptation task. First, we confirmed that motor variability on a trial-by-trial basis was selectively increased in the homogenous and prototypical dystonic disorder DYT1 dystonia. Second, high baseline variability predicted poor performance in the subsequent visuomotor adaptation task offering insight into the rules which appear to govern dystonic motor control. The potential mechanisms behind increased motor variability and its corresponding implications for the rehabilitation of patients with DYT1 dystonia are highlighted.

## Introduction

Dystonia refers to an aetiologically diverse movement disorder characterised by involuntary muscle contractions^1^. Excessive co-contraction of agonist-antagonist muscles cause abnormalities in posture and movement which are highly disabling^1^. Heterogeneity manifests at all levels of description, ranging from its clinical phenomenology (task-specific, generalised) through to cause (single gene mutations, secondary to brain lesions)^2^. Research is still far from defining a unifying pathophysiological framework for dystonia and many debate whether it should be considered a single nosological entity^2^.

One relevant line of experimental work are kinematic studies which record and analyse dystonic movements to infer which motor control mechanisms are implicated. In childhood dystonias due to mixed aetiologies there is increased motor variability^3^. This increase in trial-by-trial variability of movement is thought to be representative of increased signal-dependent noise within the motor commands produced by the dystonic brain^4,5^. Interestingly, children with dystonia can be shown experimentally to adopt strategies that minimise the impact of this variability on motor control^6^ and there is promising early work that by externally augmenting sensory feedback children are better able to suppress such variability^7,8^.

However, deciphering the significance of motor variability in disease states such as dystonia is complex. In health, for example, motor variability is also a prominent feature of behaviour and is observed when external conditions such as the sensory input or task goal are kept as constant as possible^9^. In part, variability is thought to reflect noise inherent to the nervous system, a detrimental feature to be minimised^9^. However, certain elements of variability can also be informative to the motor system. A popular example is that of young songbirds injecting ‘noise’ or variability into their song when the requirement is to optimise learning conditions, but immediately dampen such noise when high accuracy of song is required to perform to a potential mate^10,11^. Such dynamic regulation of variability is similarly observed in humans. Experimentally, individuals with greater variability of baseline movement parameters relevant to the subsequent learning task are faster learners across reinforcement and motor adaptation (error-based) task designs and the temporal structure of motor variability can be shown to shift responsively to align to the task design^12^. Therefore, in health, a proportion of motor variability facilitates motor learning representing an exploration of motor command space^13^.

Collectively these studies signify the relevance of studying motor variability in dystonia with better understanding representing a real opportunity for improving existing therapeutic options. In this study, we therefore tested patients with DYT1 dystonia, an early onset generalised form of isolated dystonia caused by a single mutation in the TOR1A gene^14^. With their homogenous genetic aetiology and ‘pure’ motor phenotype (no spasticity or other potentially confounding neurological deficits), we considered these patients an ideal group within which to examine quintessential abnormalities of motor control in dystonia^15^. Using a purpose built robotic manipulandum we designed a task to examine motor variability during reaching movements and to investigate whether this was associated with a participant’s ability to learn a novel visuomotor rotation. Given that task-relevant variability and sensorimotor adaptation are related in health^12^, an intriguing prediction is that DYT1 dystonia patients could show increased motor variability relative to healthy controls and enhanced learning during the adaptation task.

## Results

**Figure 1:**
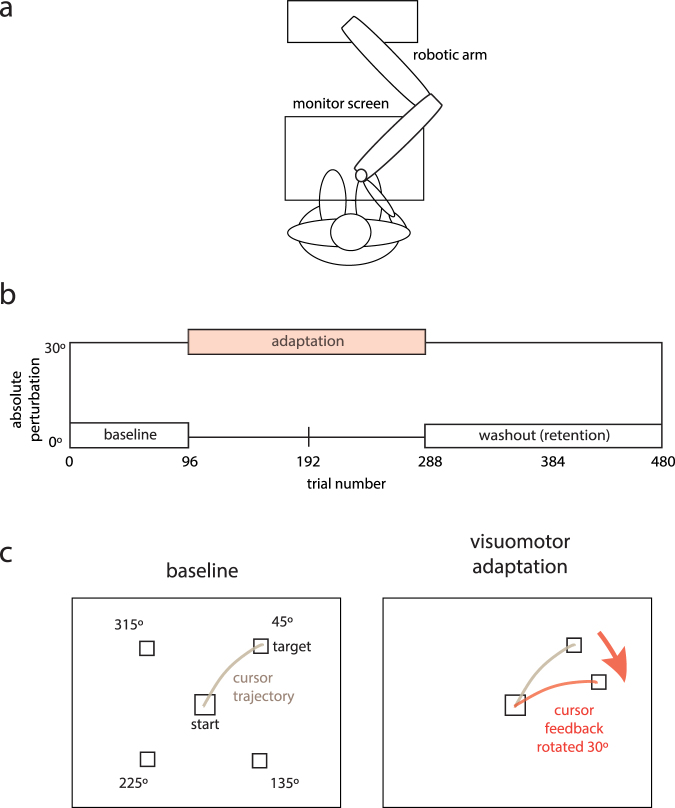
Experimental design. (**a**) Diagram of robotic manipulandum and subject. (**b**) After a baseline block with veridical online visual feedback (trials 1 to 96), adaptation to a visuomotor perturbation in which cursor feedback was rotated by 30° was tested for two blocks (trials 97 to 288). The visuomotor perturbation was then removed and retention (washout) of the rotation was evaluated with veridical online visual feedback (trials 289 to 480). (**c**) The task was displayed on a horizontal computer screen and the four potential target directions are shown. The order of target location for individual trials was pseudorandomised such that subjects made an equal number of movements to each quadrant. The visuomotor perturbation consisted of the rotation of visual feedback by 30° in either a clockwise or counter-clockwise direction.

In this study, we examined trial-by-trial variability of reaching movements in the symptomatic arm of 10 patients with DYT1 dystonia and 12 age-matched controls and explored the relationship between variability and performance in a visuomotor adaptation task. The task was displayed on a horizontal computer screen and performed using a robotic manipulandum (Fig. 1a). For every trial, participants made a fast outward movement from a central starting position towards one of four potential target positions with the aim of stopping in the target box within the fixed time frame of one second. Following a baseline block, sensorimotor adaptation was examined by applying a visuomotor perturbation of 30° (Fig. 1b,c). Veridical visual feedback was then reintroduced to examine washout (retention) of the newly learnt visuomotor rotation.

### Abnormal reach behaviour in DYT1 dystonia demonstrated during baseline

**Figure 2:**
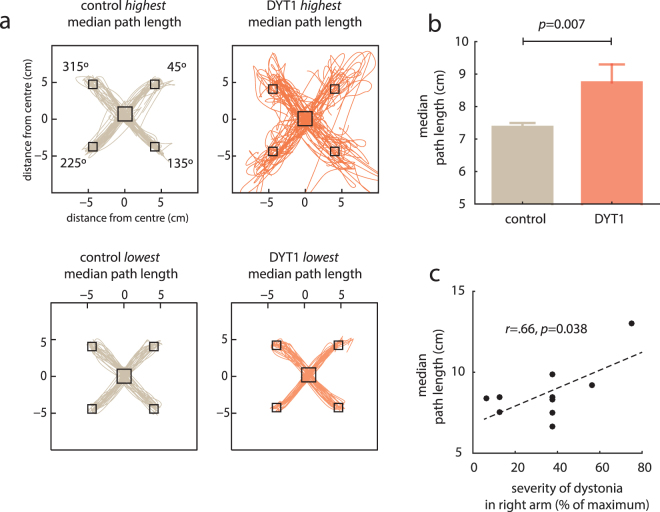
Reach behavior during the baseline block was more erratic in DYT1 dystonia with an increased median path length that correlated with the severity of dystonia. (**a**) Movements made during the baseline block of the individual with the lowest and highest median path length in the control group and DYT1 dystonia (red). Controls had a stereotyped reach strategy with little difference across trials and across individuals. In DYT1 dystonia there was a range of behaviour and subjects with increasing median path length demonstrated increasingly erratic and less efficient movements. (**b**) At the group level median path length was significantly increased in DYT1 dystonia. (**c**) In the DYT1 group, median path length was also positively correlated with the severity score of dystonia in the right arm used for the task.

We found reaching behaviour in the baseline condition to be abnormal in DYT1 dystonia. Specifically, visual differences between groups were apparent when all 96 reaches during the baseline block were plotted (Fig. 2a). To quantify such differences, we calculated path length, the total distance travelled from the start to the end of the trial. Our task was constrained by the fact that the target did not appear unless the patient was within the start box. Correspondingly there was no difference in the start error, the radial distance from the centre of the start box, between groups (baseline block, median start error controls = 0.463 cm, DYT1 = 0.461 cm, *U* = *55.0, p* = 0.742, *r* = −0.078).

Controls were stereotyped in their reach behaviour with little difference across trials, and between the individuals with the lowest and highest median path length (Fig. 2a). Conversely, in DYT1 dystonia, although some individuals had performance like controls, others had movements that were more erratic across trials and less efficient. Indeed, at the group level DYT1 dystonia was characterised by an increase in median path length (controls = 7.37 cm, DYT1 = 8.74 cm, Mann-Whitney *U* = 20.0, *p* = 0.007, *r* = −0.57, Fig. 2b). Median path length in DYT1 dystonia was also significantly related to the severity score of dystonia in the right arm (*r* = 0.66, *p* = 0.038). Therefore, we found reaching behaviour to be abnormal in DYT1 dystonia and at some level features of the task related to the phenomenology and severity of DYT1 dystonia.

### Angular accuracy, timing and force applied are unchanged in DYT1 dystonia

To better explore the elevation of path length in DYT1 dystonia, we analysed three distinct phases of movement within each trial which emphasised different motor control mechanisms (i) maximal velocity (ii) end of centre-out movement (iii) end of trial. Firstly, movement at maximal velocity is characterised by feedforward motor control, planned and executed without the integration of on-line sensory feedback^16^. The end of centre-out movement is then an approximate marker of when initial movement corrections stemming from sensory feedback start to be incorporated into behaviour^16^ and was defined by the first time velocity fell below 30% of maximal velocity. Finally, we examined movement features at the end of the trial (one second) which includes all corrective mechanisms participants have utlised (feedforward and on-line) in order to be as accurate as possible.

**Figure 3:**
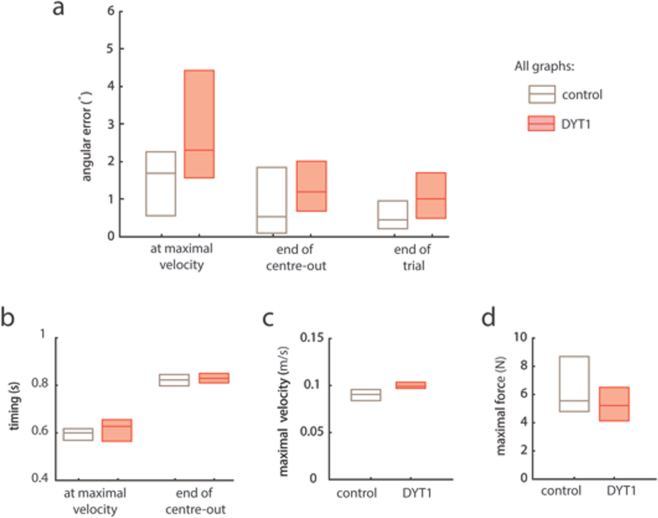
Motor deficits in DYT1 dystonia were selective: a range of accuracy, timing, velocity and force parameters were not significantly different in this basic reaching task. Median and 25^th^ and 75^th^ centiles shown by horizontal lines of box. (**a**) Although median angular error was always higher in dystonia group the ranges of error were overlapping between groups and not significantly different. (**b**) No difference in the timings of the maximal velocity peak and the end of the centre-out movement were found. End of trial was fixed at one second for all participants. (**c**) Magnitude of maximal velocity and (**d**) magnitude of maximal force applied during trial were equivalent across groups.

Interestingly, despite examining movements of a symptomatic limb, many parameters which described movement were within normal range in DYT1 dystonia. For example, evaluation of median angular error at maximal velocity, end of centre-out movement and end of trial revealed overlapping values between the groups (Fig. 3a, Supplementary Table Statistics). The timing of the maximal velocity peak and the end of the initial centre-out movement were also comparable across groups (noting that our task constrained total movement time to one second, Fig. 3b, Supplementary Table Statistics). Finally, the median magnitude of maximal velocity and maximal force applied during trials were also not different across groups (Fig. 3c,d, Supplementary Table Statistics). Together these results suggest that motor deficits in DYT1 dystonia are selective as a range of accuracy, timing, velocity and force parameters were normal in this basic reaching task.

### Increased motor variability seen throughout dystonic movements

**Figure 4:**
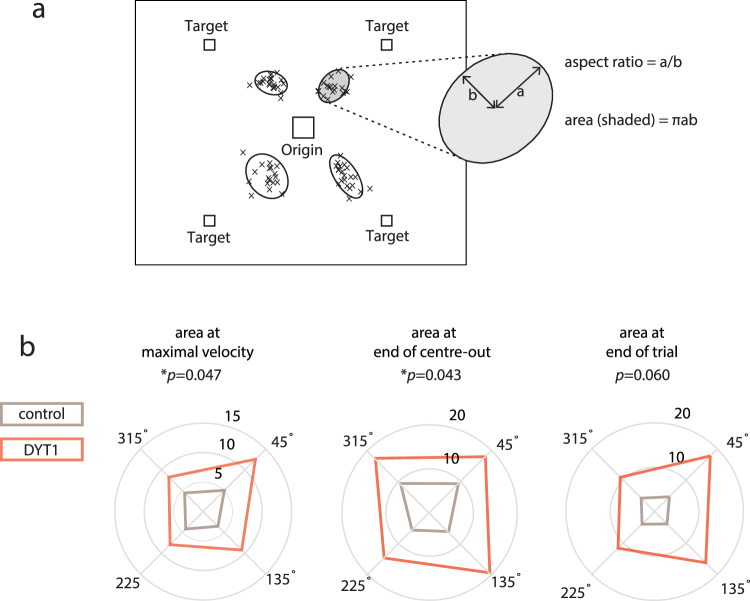
Increased spatial variability in DYT1 dystonia **(a**) Scatter of position at maximal velocity during the baseline block from an individual subject is shown. Each cross represents a trial. Confidence ellipses encompassing 95% of the variability were calculated for each reach direction and the derivatives of aspect ratio and area are illustrated. (**b**) Polar plots for area of ellipse relative to reach direction (45°, 135°, 225° and 315°). The area of the ellipse was significantly increased in DYT1 at maximal velocity and at the end of centre-out movement.

Given reports of increased variability in dystonia, we performed detailed analysis of variability features at maximal velocity, end of centre-out movement and end of trial. A scatter plot of position was drawn at each of these movement phases and in each reach direction we fitted confidence ellipses which enclosed 95% of the data points (Fig. 4a)^17^. The ellipse is a graphical representation of principal component analysis which determines the direction of the maximum and minimum dispersion of distribution in the x-y plane (the length of the axes is a function of the eigenvalues of the distribution)^17^. The aspect ratio (square root of the ratio of the two eigenvalues or larger axis divided by smaller axis) gives a measure of the shape of the ellipse. The orientation deviation is a measure of the largest eigenvalue axis relative to the target direction. The area of the ellipse gives an estimate of the total variance (pi multiplied by the axes of the ellipse). We hypothesised that the area or total variability would be increased as seen in childhood dystonias. Interestingly, the aspect ratio and orientation of the confidence ellipses were normal in DYT1 at each of three movement phases (Supplementary Table Statistics). However, as anticipated, the mean area of the ellipse, or the total spatial variance in DYT1 dystonia, was significantly increased at maximal velocity (F(1,20) = 4.50, *p* = 0.047) and the end of the centre-out movement (F(1,20) = 4.68, *p* = 0.043) (Fig. 4b). There was also a trend for this spatial variability to be increased at the end of the trial (F(1,20) = 3.97, *p* = 0.06). In summary, movements during the baseline block in DYT1 dystonia were characterised by a non-directional (normal aspect ratio, ellipse orientation) increase in motor variability.

### Task-relevant variability during baseline predicts adaptation behaviour in DYT1 dystonia

Given that task-relevant variability and sensorimotor adaptation are related in health^12^, we then examined how the increased variability in DYT1 dystonia influenced performance indicators in this visuomotor adaptation task. Markers of variability that we expected to be relevant to the task were (i) baseline angular variability at maximal velocity and (ii) angular variability at the end of trial. The baseline variability of magnitude of maximal force applied was selected as a subset of variability that was less relevant to the task. Change of hand direction at maximal velocity (adaptation) and end of trial were used as markers of performance during early and late phases of the visuomotor perturbation.

**Figure 5:**
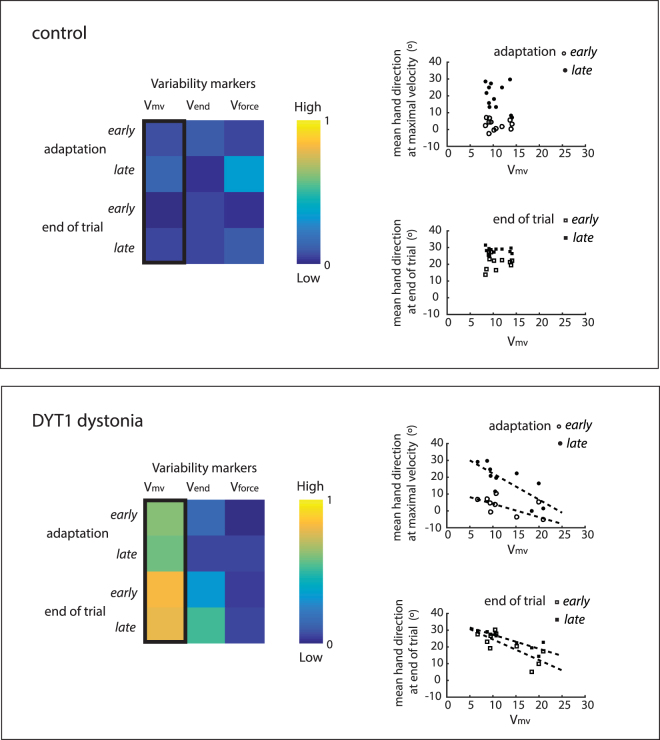
High baseline task-relevant variability in DYT1 dystonia was associated with poor performance in the sensorimotor adaptation task. (**a**) Visualisation of R^2^, the shared variance between variability markers (V_mv_, V_end_, V_force_) and performance outcomes (change of hand direction at maximal velocity (adaptation) and at the end of trial, early and late during perturbation). R^2^ has a range of possible values between 0 (no shared variance, dark blue) and 1 (all variance shared, yellow). Task-relevant variability (V_mv_) in DYT1 but not controls was significantly related to performance outcomes. Correlations of variables of interest are plotted to the right to show that high variability (V_mv_) during the baseline block in DYT1 dystonia predicted smaller changes in hand direction in response to the perturbation.

Angular variability at maximal velocity demonstrated an interesting dissociation in its relationship to performance parameters in controls and patients with DYT1 dystonia (Fig. 5). In controls, there was no correlation between angular variability at maximal velocity and performance markers (R^2^ low in Fig. 5 and all comparisons > 0.05). In contrast in DYT1 dystonia *early* adaptation (*r* = −0.789, *p* = 0.007), *late* adaptation (*r* = −0.789, *p* = 0.009), *early* end of trial hand direction (*r* = −0.903, *p* = 0.001) and *late* end of trial hand direction(*r* = −0.887, *p* = 0.001) were negatively correlated with angular variability at maximal velocity (Fig. 5, significance level = 0.0125, four comparisons).

**Figure 6:**
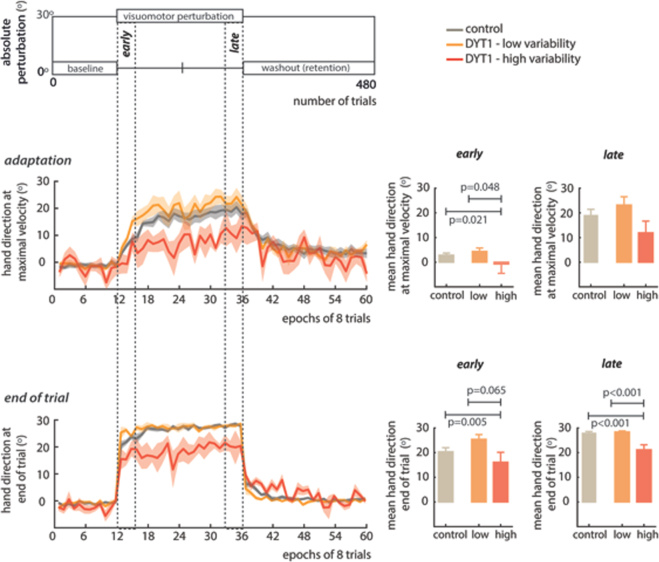
Impaired performance is seen in the high variability DYT1 dystonia group with a reduced change in hand direction both at maximal velocity (adaptation) and end of trial. A median split of DYT1 into a low and high variability group exemplifies the effects of this association. In DYT1^high^ adaptation early (significantly) and late (not significant) into the perturbation were reduced. DYT1^high^ also significantly under-achieved the optimal change hand direction at the end of trial (30°) during early and late phases of the perturbation (significantly).

A median split of patients by angular variability at maximal velocity into low and high variability groups (DYT1_low_ and DYT1^high^) illustrates this relationship further (Fig. 6). In DYT1^high^, early adaptation was reduced in comparison to both the control group and to DYT1_low_ (ANOVA between group effect: F(2,19) = 4.89, *p* = 0.019, *r* = 0.58, Fig. 6, post hoc comparisons shown in bar charts). The total magnitude of adaptation achieved late during the perturbation block was reduced in the DYT1^high^ group but not significantly (F(2,19) = 2.351, *p* = 0.122, *r* = 0.44). Hand direction at the end of trial was also significantly influenced by angular variability at maximal velocity at both *early* (F(2,19) = 6063, *p* = 0.007, *r* = 0.64) and *late* (F(2,19) = 16.9, *p* < 0.001, *r* − 0.80) stages of the visuomotor perturbation, with the high variability group showing less ability to correct for the 30*°* perturbation. Therefore, increased variability relevant to the task was negatively related to adaptation performance indicators in DYT1 dystonia.

## Discussion

In this study, we have shown that DYT1 dystonia is characterised by a selective increase in motor variability. We show how high motor variability is associated with poor adaptation performance across a range of parameters. We discuss potential mechanisms behind this increase in variability in DYT1 dystonia and the implications this has for the rehabilitation of patients with dystonia.

Motor variability can be defined as the normal variations that occur in motor performance across multiple repetitions of a task^18^. For the healthy motor system, maintaining the dynamic equilibrium of keeping unwanted variability in check whilst allowing informative elements which assist learning, is an essential regulatory role. Variability has many sources. Unwanted variability can be a consequence of noisy processes ubiquitous to the nervous system from the perception of sensory stimuli through to the generation of motor responses^9^. However, other types of variability can be beneficial. For example, Wu *et al*., showed that greater variability within baseline movement parameters relevant to the outcome of the task, predicted faster motor learning in a subsequent adaptation task^12^ (but see^19^). Moreover, features of such variability appeared to be under dynamic regulation and were modulated in response to the type of learning task^11,12^. Understanding variability is therefore complex but highly relevant in the study of movement disorders such as dystonia^18^.

In children with generalised dystonia due to heterogeneous aetiologies (such as idiopathic, genetic (DYT1), familial, neurodegenerative and secondary to brain lesions at birth) increased movement variability is a core feature^3,5,20,21^. In this study, we used a homogenous patient group with only the genetic DYT1 mutation to ensure that we were studying dystonic motor control in isolation. This was an important initial step as studies in childhood dystonias could also be influenced by additional, albeit lessor insults to the motor system, such as spasticity and weakness. It is also of interest that DYT1 dystonia classically presents in childhood after much of the normal repertoire of motor development has occurred^15^.

We found DYT1 dystonia to be characterised by a subtle increase in spatial variability. Increases in variability were maximal in phases of movement which rely on feedforward motor control (little or no influence of online feedback). Interestingly such variability appeared random in its nature with no directional preponderance shown in any of the reach conditions. This finding fits with work in children which suggests that the patterns of muscle groups or muscle synergies recruited to tasks are surprisingly intact in a disorder in which the balance between different muscle groups appears so impaired^22,23^.

Given that task-relevant variability and sensorimotor adaptation are related in health^12^, we then explored the relationship between increased variability in DYT1 dystonia and performance indicators in an error-based learning visuomotor adaptation task. We found that high task-relevant variability in DYT1 dystonia during the baseline condition predicted poor performance in the adaptation task. This result is interesting as in health, the correlation between task-relevant variability and motor learning has been plotted as a positive linear relationship suggesting that task-relevant variability is informative to the motor system^12^. If this line of reasoning is followed, one interpretation is that in DYT1 dystonia, this physiological relationship breaks down. Once an upper threshold is breached increased variability no longer assists motor learning and in DYT1 dystonia, increased variability could rather introduce error and uncertainty into the control of movement leading to the poor performance observed in this study. However, a note of caution is that we did not replicate the findings in the healthy control group by Wu *et al*. that task-relevant variability during baseline is a positive predictor for learning during sensorimotor adaptation^12^. We suspect that this may have been due to the more simplistic study design that was necessary in this patient group, however, others have questioned whether movement variability has a clear statistical relation with learning rate in health^19^.

It remains an open question what the relative importance and mechanism driving motor variability is within disease models for dystonia. One possibility is that there is an inability to remove unwanted noisy components owing to, for example, dysfunctional sensory processing or input^4^. Unwanted variability could also be secondary to a noise generator that is injecting noise at a late phase of movement preparation. One potential neuronal correlate of motor variability is the finding that there is abnormally enhanced and synchronous oscillatory activity in the output nuclei of the basal ganglia of patients with dystonia which is coherent with EMG activity during dystonic movements^24–26^. Such oscillatory activity could inject variability onto the elemental movement plan. However, dystonia is characterised by its involvement of a wide neuronal network and increased variability could be generated by multiple regions and multiple mechanisms. A better appreciation of how motor variability is generated remains an important research goal.

There is an expanding literature which advocates the role of the cerebellum in the pathophysiology of dystonia and many lines of research point specifically to cerebellar involvement in DTY1 dystonia. For example, in a recent mouse model of DYT1 dystonia expressing the gene in the cerebellum alone (but not the basal ganglia) is sufficient to induce a phenotype consistent with dystonia in humans^27^. Sensorimotor adaptation is a well-established experimental paradigm by which to examine a predominantly cerebellar function of calibrating movements in response to perturbations^28^. The data presented in this paper play an uncertain role in the cerebellar story in DYT1. Adaptation is a form or error-based learning in which the brain computes a teaching signal which is the difference between the desired movement and the actual movement (which has been perturbed by the influence of visuomotor transformation). The random spatial variability that we observed could be considered a noise factor which will be added to the teaching signal from each trial, decreasing its accuracy and certainty, and impairing the ability of the cerebellum to compute the correction or adaptation coefficient required to update the next movement. This is one very feasible explanation of our results and such an interpretation implies that cerebellar learning mechanisms themselves, per se, are not impaired in dystonia. However, in cerebellar disorders there is both increased motor execution variability and (even when accounting for this execution variability) a deficit of adaptation learning^29^. Therefore, the cerebellum may also contribute to the generation of variability, broadening the possible mechanism by which cerebellar dysfunction may be important in dystonia. Clearly, there is much further work to be done in the search to delineate the mechanisms by which the genetic mutation in DYT1 dystonia causes the movement disorder across all levels of description. It is also important to bear in mind that adaptation is known to involve cognitive strategies and multiple brain regions and cannot be considered a pure cerebellar computation^30,31^.

It is interesting to consider if there is evidence that the dystonic motor system is compensating for the increased motor variability we have observed. In general noise cannot be removed from a signal once it has been added; however processes such as averaging and weighting different components due to prior knowledge are often combined in the nervous system to counter its influence^9^. Already work in children with dystonic cerebral palsy has suggested that when learning the novel skill of throwing a virtual ball, children were able to learn and they adjusted their motor strategy to be more tolerant to variability in timing^6^. Another intriguing line of work shows that injecting increased variability into muscle feedback generates greater antagonistic muscular co-activation in order to compensate for the movement variability^32^. Increased agonist-antagonist co-activation is frequently cited as one of the hallmarks of dystonic movements^33^, and this work suggests that it could reflect a compensatory strategy.

Another important line of work which informs neuro-rehabilitory options, is that changing the sensorimotor context for patients can be helpful. Clues for this clinically may be present within sensory trick phenomena in which increased sensory feedback obtained by touching a body part (for example touching chin with hand in cervical dystonia) reduces the expression of dystonia^34^. If, as our data suggests, poor performance in DYT1 dystonia is related to increases in random variability, it is likely that the motor controller has a lesser ability to extract relevant information from actual sensory feedback as most sensory streams will be polluted by this noisy stochastic component. Therefore, externally generated and augmented feedback may offer real opportunity to reduce dystonic contractions using intact feedback loops that can improve dystonic motor control if provided additional sensory feedback. In line with this, early work has shown that visual biofeedback of muscle activity can be helpful in dystonia and scaled vibratory feedback based on muscle activation patterns can also change patterns of muscle use in children with dystonia^7,35^. Interestingly, occasionally, DYT1 dystonia can also improve with certain actions such as playing piano or knitting^36^, implying that certain motor circuits can function in a non-dystonic manner and that their activation reduces the severity of the dystonic manifestations. As with many neurological disorders there remains an unmet clinical need to develop rehabilitative strategies which exploit intact sensorimotor learning^7^. By reverse engineering dystonic control mechanisms and utilising intact features of the motor controller there is an optimistic future for targeting therapeutic interventions which counter variability in DYT1 dystonia.

Our study was limited by the small number of patients available to study due to the rarity of patients with DTY1 dystonia in adult life that have not already been treated by deep brain stimulation. There is also the potential confound that some patients were taking medications. However, we considered the potential difficulties patients can endure coming off their long-term medications to be too disabling and note that no patients reported side effects from the medications such as cognitive symptoms.

In conclusion, we have shown that patients with the prototypical dystonic disorder, DYT1 dystonia, have movements characterised by a selective increase in the spatial variability of movements. High levels of variability were significantly associated with reduced learning performance during sensorimotor adaptation. Determining causal mechanisms of the excess motor variability in DYT1 remains an important research aim.

## Methods

### Subjects

Ten patients with generalised DYT1 dystonia and 12 age-matched controls were recruited (Table 1). Those receiving botulinum toxin injections were tested at the end of their therapeutic window (minimum three months post last injection) and none had received deep brain stimulation. Subjects had no additional neurological/musculoskeletal problems of the arm or significant cognitive impairment. Both groups were novices to the robot and the motor task. The study had been approved by the local ethics committee and was carried out in accordance with the Declaration of Helsinki (NRES Committee London - Camden & Islington, REC reference: 11/LO/0307). Written informed consent was obtained from all participants.

**Table 1:** Patient characteristics.

	Age	Sex	Hand	Arm Severity	Total Severity	Duration Dystonia	Medication
DYT1							
	24	F	R	6	16	15	BTX (forearms)
	24	F	R	6	32	2	trihexyphenidyl, clonazepam
	33	F	R	6	16	22	BTX (bilateral lower limb,not administered >1 year)
	42	M	R	1	8	16	trihexyphenidyl
	44	F	R	2	29	34	trihexyphenidyl, clonazepam
	46	M	R	2	15	13	BTX (paraspinal)
	48	F	R (L)	6	14	40	trihexyphenidyl, clonazepam
	50	M	R (L)	12	55	42	trihexyphenidyl, BTX (not administered >1 year),
	59	M	R	6	38	50	diazepam, baclofen, BTX (paraspinal)
	69	F	R (L)	9	46	58	trihexyphenidyl, clonazepam
mean (SD)	43.9 (±14.3)						
Controls							
mean (SD)	42.3 (±13.8)						

Hand preference is documented at the time of the study (if different, hand preference during childhood is given in brackets). The duration of symptoms (from onset to current age) is given in years. The Fahn-Marsden Motor Score for the right arm (maximum severity = 16) and total score (maximum severity = 120) are detailed. Site of botulinum toxin injections (BTX) indicated in brackets. There was no significant difference between patients and controls in respect to age *t*(20) = −0.223 *p* = 0.826.

### Task

Participants were seated with their forehead supported on a headrest. Their right hand gripped the robotic manipulandum underneath a horizontally suspended mirror. The robotic arm had two joints that allowed fluid movement along a horizontal plane. The mirror prevented direct vision of the arm and showed the reflection of a computer monitor mounted above (Fig. 1a). The central starting position was marked by a white box of diameter 1.5 cm and the position of the manipulandum was indicated by a white circle of radius 0.3 cm. For each trial to be initiated the cursor had to be within the central starting square. A white target box of diameter 1 cm subsequently appeared in a pseudorandomised order in one of four radial locations 6 cm from the centre (directions: 45°, 135°, 225°, 315°). Subjects were instructed to make a fast reaching movement towards the target square and to stop within the target as accurately as possible (Fig. 1b). Subjects received visual feedback during the active movement. The movement time was set at one second at which point a green circle appeared to mark the end position of the trial. At the end of each trial, the robot (passive movement, patient asked to relax) returned the hand/manipulandum back to the central starting position, without visual feedback.

Participants familiarised themselves with the robot and task by performing 25 trials during which verbal feedback was given to further explain the desired movement (data not analysed). Each subject then completed three different experimental conditions: a baseline block consisting of 96 trials, an adaptation block of 192 (2 × 96) trials and a washout block of 192 trials (Fig. 1b). In the baseline block, targets appeared in a pseudorandomised order. In the adaptation block, presentation of targets was as for the baseline block, but a constant 30° rotation in the clockwise (positive) or anticlockwise (negative) direction was applied to the screen cursor (representing the subject’s hand position). The direction of the perturbation was randomised across subjects. In the washout block, the perturbation was removed. The approximate total time of the experiment was 25 minutes.

### Analysis

The 2-D position of the cursor was sampled at a rate of 100 Hz. Analysis was run using custom written matlab scripts (Matlab R2015a, TheMathWorks). Units used throughout are centimetres (cms), degrees (°), milliseconds (ms), meters per second (m/s) and Newtons (N). Movement parameters were calculated for each trial and median and standard deviation across the baseline block were used to compare central tendency and the variability. *Start error* was defined as the radial distance from the centre of the start box when the target appeared. *Path length* was the total distance travelled from the start to the end of the trial and is elevated with any non-efficient deviation of trajectory from start to finish. The three different phases of movement analysed in each trial were: (i) maximal velocity within trial (ii) end of centre-out movement (first time velocity fell below 30% of maximal velocity after maximal velocity) and (iii) end of trial. *Angular error* was the deviation in degrees from the direct path which links the start point to target at these three time points. In addition, the *timings* of the maximal velocity peak, end of centre-out movement, *maximal velocity* magnitude and *maximal force* magnitude applied to manipulandum were noted for each trial. To avoid sensitivity to outliers, any trials in which the total radial distance travelled was <2 cm (incomplete movement), or the angular error was greater than 60° in either a clockwise or anticlockwise direction at maximal velocity or the end of the trial (likely error in identifying target reach direction) were excluded. This excluded 1.47% of trials in controls, and 1.58% of trials in subjects with DYT1 dystonia.

To investigate the spatial distribution of variability during the baseline block, 95% confidence ellipses of the scatter of cursor position (x, y) were calculated at each of these movement phases. The ellipses are obtained by applying a principal component analysis to determine the direction of maximum and minimum dispersion of distribution in the x-y plane^17,37^. The eigenvectors of the covariance matrix are the axes of the ellipse, while the lengths of the axes are the corresponding eigenvalues. We calculated three parameters to fully describe each ellipse. The *aspect ratio* was the square root of the ratio of the two eigenvalues (the larger divided by the smaller) as a measure of the shape of the ellipse. The *orientation deviation* was the orientation of the largest eigenvalue relative to the target direction. Since this measure has low reliability for distributions that are approximately circular we multiplied the orientation deviation by the (aspect ratio-1) which weights each data value by its reliability^17^. The total variance was estimated by the *area* of the ellipse (pi multiplied by the axes of the ellipse).

Learning and retention were assessed during the application and removal of the visuomotor transformation respectively^38^. Extent of adaptation learning can be estimated by examining the change of hand direction at maximal velocity (deviation in degrees from the direct path^16^) and as the perturbation is learnt increasing adaptation is seen which approaches the 30° transformation. Extent of ‘*early’* adaptation was estimated by the mean hand direction of trials 2 to 17 from the onset of the perturbation. ‘*Late’* adaptation was the mean hand direction of last 16 trials before visuomotor transformation was removed. Memory for adaptation, *retention*, was the mean hand direction for the first 16 trials after the perturbation had been removed. In addition, change of hand direction at the end of trial was noted, during ‘early’ and ‘late’ trials of the perturbation, and is composite metric of both adaptive and online corrective processes to counter the 30° visual transformation.

To examine the relationship between variability and performance we selected markers of variability during baseline that were likely to be relevant to the subsequent visuomotor adaptation task: (i) angular variability at maximal velocity^12^ and (ii) angular variability at the end of the trial. Variability of maximal force magnitude across trials during the baseline block was used as a marker of variability which was less relevant to the task in which a 30° visual transformation is introduced.

Due to the small sample size we used the non-parametric Mann-Whitney U test to compare the two groups (test statistics (*U)*, its significance (*p*) and the effect size (*r))*. Repeated measures analysis of variance (rmANOVA) was used to compare confidence ellipses parameters across the four reach directions (repeated factor) with group as a between subject factor (F-statistic (*F)*, significance *(p)*). One-way ANOVA with three subject groups (control, DYT1_low_ and DYT1^high^) was used to compare mean values of adaptation, end error, online learning, retention and change in baseline variability (F-statistic (*F)*, significance *(p)* and effect size *(r)*). Bonferonni’s adjustment was used for posthoc pairwise comparisons. Bivariate correlations were used to assess for covariance between parameters, all correlations were two-tailed. IBM SPSS Statistics v24 was used for all statistical analysis.

## Electronic supplementary material

Supplementary Table
