# Supplementary material for: High motor variability in DYT1 dystonia is associated with impaired visuomotor adaptation
Source: Sci Rep. 2018 Feb 26;8:3653. doi: 10.1038/s41598-018-21545-0 (PMC5826938; doi:10.1038/s41598-018-21545-0)
Supplement: Supplementary file 1 — Supplementary Table [file 41598_2018_21545_MOESM1_ESM.docx]

### Supplementary information for manuscript:

### High motor variability in DYT1 dystonia is associated with impaired visuomotor adaptation

*Anna Sadnicka, Anna Stevenson, Kailash P Bhatia, John Rothwell, Mark J Edwards, Joseph M. Galea*

**Table.** **Statistical Results**. Mann-Whitney U test was used to compare the two groups (test statistics (*U)*, its significance (*p*) and the effect size (*r))*. Repeated measures analysis of variance (rmANOVA) was used to compare confidence ellipses parameters across the four reach directions (repeated factor) with group as a between subject factor (F-statistic (*F),* significance *(p)*).

| **Parameter** | **Result** |
| --- | --- |
| **Baseline**  *Angular error (degrees*) |  |
| Maximal velocity | *U*=34.0, *p*=0.093, *r*=-0.365 |
| End of centre-out | *U*=40.0, *p*=0.203, *r*=-0.279 |
| End of trial | *U*=30.0, *p*=0.059, *r*=-0.407 |
| *Timing (seconds)* |  |
| Maximal velocity | *U*=44.5, *p*=0.314, *r=*-0.218 |
| End of centre-out | *U*=54.5, *p*=0.722, *r=*-0.078 |
| *Maximal velocity* | *U*=34.0, *p*=0.093, *r*=-0.365 |
| *Maximal force exerted* | *U*=44.0, *p*=0.314, *r*=-0.225 |

| **Confidence ellipse /**  **principle component analysis**  *Aspect ratio* |  |
| --- | --- |
| Maximal velocity | F(1,20)=.323, *p*=0.576 |
| End of centre-out | F(1,20)=.714, *p*=0.408 |
| End of trial | F(1,20)=1.72, *p*=0.202 |
| *Orientation deviation* |  |
| Maximal velocity | F(1,20)=2.83, *p*=0.108 |
| End of centre-out | F(1,20)=3.55, *p*=0.074 |
| End of trial | F(1,20)=0.58, *p*=0.812 |
| *Area* |  |
| Maximal velocity | F(1,20)=4.50, **p*=0.047 |
| End of centre-out | F(1,20)=4.68, **p*=0.043 |
| End of trial | F(1,20)=3.97, *p*=0.06 |
